# *Minichromosome Maintenance Complex (MCM)* Genes Profiling and *MCM2* Protein Expression in Cervical Cancer Development

**DOI:** 10.31557/APJCP.2019.20.10.3043

**Published:** 2019

**Authors:** Gurjeet Kaur, Shandra Devi Balasubramaniam, Yung Jen Lee, Venugopal Balakrishnan, Chern Ein Oon

**Affiliations:** *Institute for Research in Molecular Medicine, Universiti Sains Malaysia, 11800 Minden, Pulau Pinang, Malaysia. *

**Keywords:** Minichromosome maintenance complex, MCM- cervical carcinogenesis, gene expression

## Abstract

**Objective:**

Minichromosome maintenance complex (MCM) proteins are essential for the process of DNA replication and cell division. This study aimed to evaluate *MCM* genes expression profiles and MCM2 protein in HPV-associated cervical carcinogenesis.

**Methodology:**

*MCM2*, *4*, *5* and *7* genes expression profiles were evaluated in three cervical tissue samples each of normal cervix, human papillomavirus (HPV)-infected low grade squamous intraepithelial lesion (LSIL), high grade squamous intraepithelial lesion (HSIL) and squamous cell carcinoma (SCC), using Human Transcriptome Array 2.0 and validated by nCounter^®^ PanCancer Pathway NanoString Array. Immunohistochemical expression of MCM2 protein was semi-quantitatively assessed by histoscore in tissue microarrays containing 9 cases of normal cervix, 10 LSIL, 10 HSIL and 42 cases of SCC.

**Results:**

*MCM2*, *4*, *5* and *7* genes expressions were upregulated with increasing fold change during the progression from LSIL to HSIL and the highest in SCC. *MCM2 *gene had the highest fold change in SCC compared to normal cervix. Immunohistochemically, MCM2 protein was localised in the nuclei of basal cells of normal cervical epithelium and dysplastic-neoplastic cells of CIN and SCC. There was a significant difference in MCM2 protein expression between the histological groups (P = 0.039), and histoscore was the highest in HSIL compared to normal cervix (P = 0.010).

**Conclusion:**

The upregulation of *MCM* genes expressions in cervical carcinogenesis reaffirms MCM as a proliferative marker in DNA replication pathway, whereby proliferation of dysplastic and cancer cells become increasingly dysregulated and uncontrolled. A strong expression of MCM2 protein in HSIL may aid as a concatenated screening tool in detecting pre-cancerous cervical lesions.

## Introduction

Cervical cancer is ranked the fourth most common cancer among females and the eighth most common cancer gobally according to the World Health Organization and International Agency for Cancer Research (IARC) (GLOBOCAN, 2018). High risk human papillomavirus (HPV) including HPV 16 and 18 are the main etiological agents involved in cervical cancer development (Zur Hausen, 2002). The viral oncoproteins E6 and E7 interact and degrade *p53* and *RB* tumour suppressor genes in the host cells. This causes increased cell proliferation and reduction in apoptosis, leading to immortalization of cells and development of cancer (Zur Hausen, 2002). 

The family of minichromosome maintenance complex (MCM) proteins (MCM2–7 and MCM10) are essential for initiating the process of DNA replication and cell division (Bell and Dutta, 2002; Bochman and Schwacha, 2009). The MCMs act as replicative helicases, and together with CDC6 and CDT1, are components of the pre-replicative complex (Bell and Dutta, 2002; Bochman and Schwacha, 2009). Additional factors, such as cyclin-dependent kinase (CDK) and polymerase are recruited to activate DNA unwinding and initiate DNA replication during S phase. Throughout late S, G2 and early M phases, CDKs cause dissolution of these components, thereby making DNA replication a ‘once per cycle’ affair. MCMs are markers for proliferation, evidenced by high activity in proliferating cells, and down-regulation in quiescence or in cells undergoing differentiation (Freeman et al., 1999; Madine et al., 2000). Abnormalities in its function could result in chromosomal defects that may contribute to tumorigenesis. 

The MCM family members have a DNA-dependent ATPase motif in the central domain. MCM4, 6, and 7 form a hexameric complex and function as a DNA helicase in vitro (Tanaka et al., 1997). This suggests that MCM4, 6 and 7 complex acts as a DNA-unwinding enzyme during replication. In contrast, MCM2, 3, and 5 proteins were found to inhibit the helicase activity of MCM 4, 6, and 7 by disassembling this hexamer (Sato et al., 2000). In vivo findings indicate that MCM2-7 proteins act as a replicative helicase that is responsible for fork movement (Labib et al., 2000). Therefore, MCM2-7 complex is likely to be involved in DNA replication as a helicase. 

MCMs are candidate markers for cell proliferation and increased levels of MCMs indicate proliferation of malignant cells. Moreover, some evidence suggests that MCMs predict tumour progression. Studies have indicated that MCM proteins are highly expressed in several types of cancers, such as lung, breast, colon, and other cancers (Shetty et al., 2005; Giaginis et al., 2009; Liu et al., 2017). They may also be useful as potential diagnostic or prognostic markers (Giaginis et al., 2010). 

MCM2 has been recognised as a useful marker in screening for cervical carcinoma (Amaro Filho et al., 2014) oral squamous cell carcinoma (Razavi et al., 2015) and medulloblastoma (Jin et al., 2014). MCM3 was identified as a better indicator for the evaluation of dysplastic oral lesions compared to Ki-67 (Lameira et al., 2014). In addition, mutation of MCM4 was detected in skin cancer cells, that affects the DNA helicase activity of the MCM2-7 complex (Ishimi and Irie, 2015). Furthermore, MCM5 was associated with breast cancer prognosis (Eissa et al., 2015), while MCM7 may be useful in predicting the prognosis of papillary urothelial neoplasia (Guan et al., 2015). 

HPV infection is normally cleared by the immune system but if an infection persists in the cervical epithelium, the host and viral genomes undergo alterations that cause cervical cells to transform into a distinctive pre-cancerous stage termed squamous intraepithelial lesion (SIL), and further into invasive squamous cell carcinoma (SCC). The main reason for the persistence of infection is due to expression of HPV E6 and E7 proteins in host cells which cause increased levels of cell cycle proteins such as p16, Ki-67 and MCM (Pett and Coleman, 2007; Doorbar et al., 2015; Graham, 2017). p16 is considered a key tissue biomarker in determining the increased HPV E6 and E7 activity in pre-cancerous lesions and cancer of the cervix (Reuschenbach et al., 2014). MCM, a cellular marker of DNA replication, has also been established as an alternative biomarker (Griffin et al., 2015). Carcinogenesis is inextricably linked with loss of cell cycle regulation and abnormal DNA replication. Several studies showed increased MCM2 protein expression in HPV-infected cells (Amaro Filho et al., 2014; Zheng, 2015) and cervical cancer (Das et al., 2013; Amaro Filho et al., 2014). Very few studies have focused on pre-cancerous lesions, with variable results (Nicol et al., 2012; Saritha et al., 2018). 

Therefore, this study provides a unique advantage to study the expression of *MCM* through the process of cervical carcinogenesis. This study aimed to evaluate *MCM2*, *4*, *5* and *7* genes expression profiles and their putative role in HPV-associated cervical carcinogenesis. Further assessment of MCM2 protein was performed to investigate it’s potential as a marker in detecting pre-cancerous lesions and cervical cancer. 

## Materials and Methods

*Tissue samples*

Histologically confirmed cases of normal cervix, low-grade squamous intraepithelial neoplasia (LSIL), high-grade squamous intraepithelial neoplasia (HSIL) and squamous cell carcinoma (SCC) were selected from the hospital pathology records. Patients’ demographic data remained confidential and only the pathology report was acquired. The formalin-fixed paraffin-embedded (FFPE) cervical tissue samples comprised biopsies, conization and hysterectomy specimens. The LSIL, HSIL and SCC samples were pre-determined to be HPV type 16 and/or 18 status by immunohistochemistry and real time PCR. Only HPV 16/18 positive cases of SIL and SCC were included in the study. Normal cervical tissues were obtained from hysterectomy specimens removed for other non-cervical related conditions. They were confirmed HPV negative and served as negative control. The pathology diagnoses were reviewed by a pathologist (Gurjeet Kaur). Each histological group comprised of three cases. Approval was obtained from the Human Research Ethics Committee of Universiti Sains Malaysia (USM/JEPeM/279.3 (1)). 

*RNA extraction*

Gene expression studies were performed on twelve samples; 3 normal cervix, 3 each of HPV 16/18-positive LSIL, HSIL and SCC. Laser capture microdissection was used to isolate the pathological lesion from the tissue sections. RNA extraction was done on the micro dissected tissues using RNeasy FFPE extraction kit (Qiagen, Germany) according to the manufacturer’s protocol. The purity of the RNA was measured using Nanodrop and quantified using a bioanalyser (Agilent, USA). 

*Human Transcriptome Array 2.0*

GeneChip Human Transcriptome Array 2.0 (HTA 2.0) Affymetrix, USA was used to profile the gene signatures involved in the disease progression of cervical cancer. This study focused on the expression profile of MCM family genes, comprising *MCM2*, *4*, *5* and *7*. Sensation plus FFPE WT kit (Affymetrix, USA) was used for all 12 samples following the manufacturer’s protocol. Upon hybridization, the chip was scanned using Affymetrix GeneChip Scanner 3000. The data was analysed by Affymetrix GeneChip Operating Software (GCOS), which contains qualitative and quantitative analysis for every probe set. Affymetrix^®^ Transcriptome Analysis Console (TAC) software was used to determine the expression profile of MCM genes family in each histological group compared to normal cervix. 

*nCounter® PanCancer Pathway Array*

To validate the differentially expressed *MCM2*, *4*, *5* and *7* genes, nCounter^®^ PanCancer Pathway Array (NanoString, USA) was used according to the manufacturer’s protocol for mRNA expression profiling, comparing the gene expression in each histological group to normal cervix. The data was analysed with nSolver 3.0 software (NanoString, USA). 

*Evaluation of immunohistochemical expression of MCM2 protein in tissue microarrays*

Two FFPE tissue microarrays (TMA) (BB10011 and CR1003, Biomax USA) were used for MCM2 protein evaluation containing 10 cases of normal cervix, 10 LSIL, 10 HSIL and 43 SCC cases, with two identical tissue cores per case. HeLa cell block was used as the positive control. Briefly, HeLa cells were washed with phosphate-buffered saline and centrifuged at high speed to remove the supernatant. After fixation of the cell pellet in 4% formaldehyde for 24 hours at 4oC, the sample was centrifuged at 1800 RPM for 10 minutes. Then the pellet was incubated in 70% ethanol for 30 minutes at room temperature, followed by centrifugation at 1800 RPM for 10 minutes. The supernatant was removed. The cells were incubated overnight in 100% ethanol at 4^o^C. Following that, the fixed cells were centrifuged at 1,800 RPM to remove the supernatant. The cell pellet was folded in lens paper and placed in a cassette for routine tissue processing, paraffin embedding and sectioning. 

The TMAs and positive control tissue were subjected to immunohistochemical staining using a standard protocol. Briefly, antigen retrieval was performed with citrate buffer (10mM, pH 6.0) and heated in a microwave oven for 20 mins at low setting. After treatment with peroxidase-blocking solution (Dako, USA), sections were blocked with 5% bovine serum albumin (Sigma-Aldrich, USA) and 5% goat serum (Biowest, France) in Tris-buffered saline-Tween-20 (TBS-T) solution at room temperature for 1 hour. Primary rabbit monoclonal anti-MCM2 antibody (Abcam, UK, 1:50 dilution) was applied and incubated overnight at 4oC. Biotinylated secondary antibody, goat anti-rabbit, (Abcam, UK), 1:500 was added, followed by avidin-biotin complex, Vectastain ABC reagent (Vector Laboratories, USA) for one hour each. Slides were rinsed thrice with TBS-T between the incubation steps. Finally, DAB (3.3′-diaminobenzidin) chromogen (Dako, USA) was added and allowed to develop for 10 mins, rinsed and counterstained with Mayer’s haematoxylin (Dako, USA). Under light microscope, brown staining cervical epithelium nuclei signified positive protein expression. The TMA slides were scanned using a Hamamatsu, NanoZoomer S60 Digital Slide Scanner. The histoscore was calculated by multiplying the percentage of positivity score and staining intensity score, shown in Table 1. 

*Statistical Analysis*

The association between MCM2 protein expression and histological groups were analysed using chi-square test, with statistical significance set at p <0.05, using IBM SPSS V24.0 software package for Windows. 

## Results

Human Transcriptome Array 2.0 was performed to determine the *MCM2*, *4*, *5* and *7* genes expression profiles in twelve FFPE samples of normal cervix, HPV-associated LSIL, HSIL and SCC. The results were validated using the NanoString platform. Following that, immunohistochemical expression of MCM2 protein was evaluated in 73 cases of cervical lesions contained in two tissue microarrays. 

**Figure 1:**
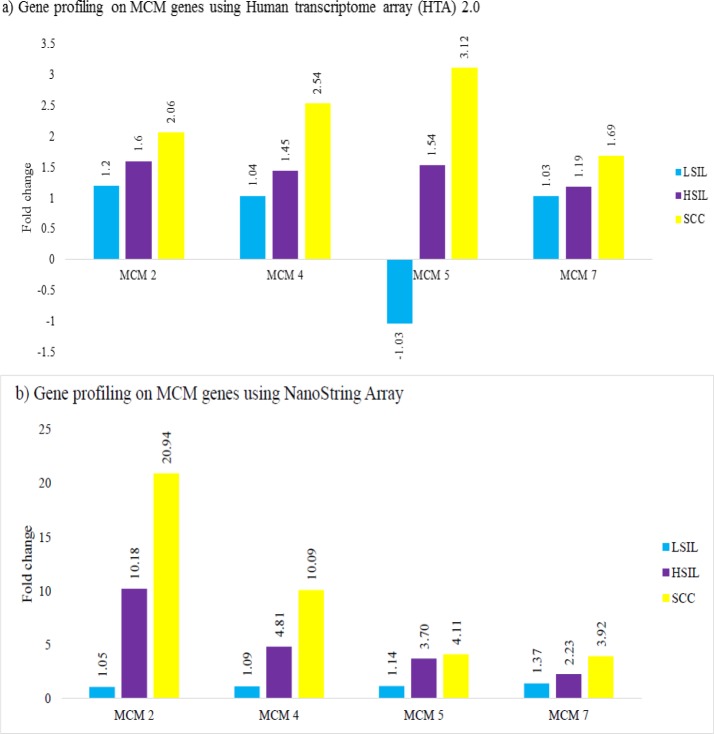
Gene Expression Profile of MCM Gene Family in Histological Groups of Low-Grade Squamous Intraepithelial Lesion (LSIL), High-Grade Squamous Intraepithelial Lesion (HSIL) and Squamous Cell Carcinoma (SCC), each Group Compared to Normal Cervix. Results were obtained from (a) HTA and (b) NanoString pan-cancer pathway. Bars represent the fold change difference in each histological group compared to normal cervix

**Figure 2:**
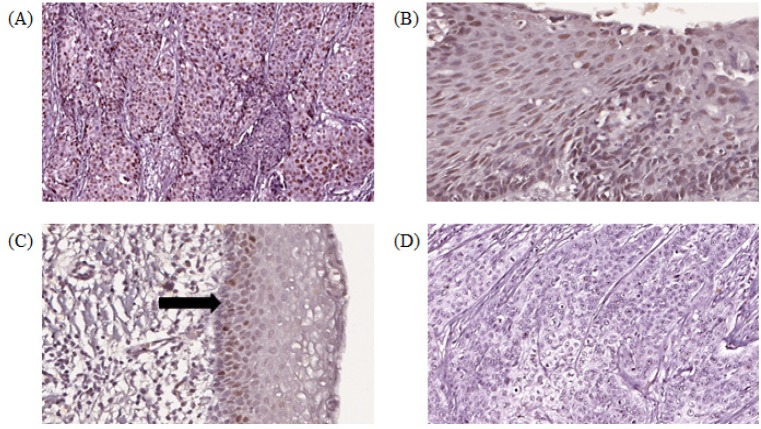
Digital Scanned Images of MCM2 Immunohistochemical Staining Showing Brown Nuclear Staining in Cervical Epithelial Cells. (A) squamous cell carcinoma (SCC) showing high histoscore, (B) high-grade squamous intraepithelial lesion (HSIL) with high histoscore, (C) Normal cervix with low histoscore, positive staining in basal cells (arrows), (D) squamous cell carcinoma (SCC) with negative expression (objective x 20)

**Table 1:** Histoscore Method for Immunohistochemical Expression

Percentage of positivity	Score	Staining intensity	Score
< 1% positive cells	0	No staining	0
1% - 25% positive cells	1	Weak staining	1
26% - 50% positive cells	2	Moderate staining	2
51% - 75% positive cells	3	Strong staining	3
≥ 75% positive cells	4		

Final histoscore, Percent positivity score x Staining intensity score; Score : 0, Negative; 1 – 3/12, Low expression; ≥ 4/12, High expression

**Table 2:** MCM2 Histoscore in Normal Cervix, LSIL, HSIL and SCC, and Comparison between Individual Histological Groups and Normal Cervix

Histological	MCM2 histoscore			Total		Histological group vs normal
group	Negative n (%)	Low n (%)	High n (%)	n (%)	p value	p value
Normal	3 (33.3)	4 (44.4)	2 (22.2)	9 (100)		
LSIL	1 (10.0)	3 (30.0)	6 (60.0)	10 (100)		0.212
HSIL	0 (0.0)	1 (10.0)	9 (90.0)	10 (100)	0.039	0.01
SCC	13 (31.0)	14 (33.3)	15 (35.7)	42 (100)		0.713
Total	17 (23.9)	22 (31.0)	32 (45.1)	71 (100)		

Chi-square test, statistical significance set at p value <0.05; LSIL, low-grade squamous intraepithelial lesion; HSIL high-grade squamous intraepithelial lesion; SCC, squamous cell carcinoma.

*Gene expression profiling using Human Transcriptome Array (HTA 2.0) and NanoString*

MCM genes expressions were determined in LSIL, HSIL and SCC groups, compared to normal cervix, using Human Transcriptome Array (HTA2.0) and validated by NanoString nCounter^®^ PanCancer Pathway Array. In general, there was increase in fold change of *MCM2*, *4*, *5* and *7* genes during the progression from LSIL to HSIL with the highest fold change in SCC (Figure 1). NanoString data showed concordance in trends of expression except for LSIL whereby MCM5 gene was downregulated in HTA but upregulated in NanoString. In NanoString analysis, *MCM2*, *4*, and *7* genes were upregulated in HSIL and SCC. MCM2 gene had the highest fold change in SCC compared to normal cervix.

*MCM2 immunohistochemical expression*

Histoscore was assessed on 42 cases of SCC, 10 LSIL, 10 HSIL and 9 normal cervix in the TMAs. One tissue core of SCC and normal cervix did not contain squamous epithelium, hence excluded from the study. MCM2 protein was expressed in nuclei of cervical epithelial cells and HeLa cells. Stromal inflammatory cells showed both nuclear and cytoplasmic staining. In normal cervix, nuclear staining was observed in basal and parabasal cells, whereas in SIL, dysplastic cells occupying varying degrees of epithelial thickness were positively stained. The photomicrograph images of MCM2 histoscore expressions are shown in Figure 2.

The histoscore results of MCM2 protein expression are depicted in Table 2. Overall, *MCM2* expression was positive in 54 (76.1%) of 71 cases. The highest expression was noted in HSIL group. Chi-square test demonstrated that there was a significant difference in *MCM2* expression between the histological groups (P = 0.039), and between HSIL and normal cervix (P = 0.010). 

## Discussion

During cervical neoplastic transformation, cells acquire the ability to sustain proliferation and resist cell death or apoptosis. The HPV viral oncoproteins, primarily the E6 and E7, dysregulate the normal function of host’s tumour suppressor genes *p53* and *pRb*, causing the cells to enter the S phase and continue proliferation. This event eventually leads to abnormalities in cell growth and function. Progression through the cell cycle requires expression of genes that regulate the cell cycle check points (G1 and G2). Up-regulation of CDKN2A, also known as p16, in the early stage of cervical cancer is an indication of the host response in inactivating pRb gene and releasing the E2F family (Gius et al., 2007). The interaction between high risk HPV types 16/18 and p16 appears to play a major role in cervical carcinogenesis. 

DNA replication is a key process in cell proliferation and cell growth. The abnormal proliferation of tumour cells is characterized by irregularities in pathways involved in DNA replication, cell cycle, and apoptosis. A series of events that occurs during HPV infection causes the host cells to undergo unscheduled cell cycle. This phenomena leads to uncontrolled cell division and enhanced cell proliferation, further causing cancer development. In HPV-associated cervical cancer, the cancer cells upregulate specific genes that control several steps in DNA replication (Cheng et al., 2017). MCMs have been identified as the key regulators in DNA replication, involved in the helicase activity for fork movement.

The MCM proteins are markers of cell proliferation and have been shown to be highly expressed in a variety of cancers including breast, lung, colorectal and many other types of cancers (Shetty et al., 2005; Giaginis et al., 2009; Liu et al., 2017). Though studies have reported on MCM expression in cervical cancer, very few have analysed the whole spectrum of transformation of HPV-infected cervix into pre-cancerous lesions (LSIL and HSIL) and further into invasive cancer (Das et al., 2013; Niu et al., 2017; Li et al., 2018; Saritha et al., 2018). This study aimed to evaluate the *MCM mRNA* expressions in HPV-associated pre-cancerous and cervical cancer tissues, to better understand their role in cervical carcinogenesis. Moreover, the protein expression of MCM2 was also determined in this study. 

Our results on transcriptomic profiling using both Human Transcriptome Array (HTA) and NanoString clearly demonstrated an upregulation and increasing fold change of *MCM2*, *4*, *5* and *7* genes during the progression from LSIL to HSIL and SCC, compared to normal cervix. The highest fold change was in the SCC group. The results in our study concur with others that report an upregulation of* MCM2*, *4* and *5* genes in cervical SCC compared to normal samples (Niu et al., 2017; Li et al., 2018). MCM2, 4, 5,6 and 7 were also found to be upregulated in cervical cancer using semi-quantitative RT-PCR technique (Das et al., 2013). Pre-cancerous lesions were not in the scope of these studies. In LSIL, dysplastic cells are limited to the lower one third of cervical epithelium, reflecting a low number of cells in the proliferative phase. It is also well recognised that a high percentage of LSIL cases can revert to normal when HPV infection is cleared by host immunological mechanisms (Hausen, 2002). Therefore, the genomic signature of LSIL would understandably be quite similar to normal cervix, demonstrated by approximately one-fold change in the *MCM* genes. In *HSIL*, the *MCM* gene family expression is upregulated as the abnormal dysplastic cells acquire genetic alterations that promote cell growth and survival. As the disease progresses into cancer, there is uncontrolled cell division and DNA replication, reflected by a further increase in MCM gene expression. 

Interestingly, MCM2 showed the highest upregulation in SCC compared to normal cervix, from the NanoString data. When comparing both methods of transcriptomic profiling, it was evident that HTA showed a lower gene expression fold change compared to NanoString. This is related to the distinct types of expression detection systems that give superiority to NanoString (Geiss et al., 2008; Tsang et al., 2017). In NanoString, the direct measurement of mRNA expression levels using multiplexed, colour-coded probe pairs without the need for amplification is considered more sensitive than microarrays and similar in sensitivity to real-time PCR (Geiss et al., 2008). Studies have reported that results from NanoString are concordant to the HTA platform (Zhu et al., 2016; Delmonico et al., 2019). Furthermore, NanoString is found to be suitable for accurately measuring mRNA transcripts in archival FFPE-derived biological samples, which are acknowledged to have poor quality and low yield of RNA (Reis et al., 2011; Tsang et al., 2017). Upregulation of MCM2 at mRNA levels prompted us to focus on the expression of MCM2 at protein level and to identify it’s potential as a biomarker in screening for cervical carcinoma, as previously reported by Amaro Filho et al., (2014).

Histologically, *MCM2* expression was restricted to the nuclei of basal and parabasal layers of normal cervix squamous epithelial cells in the tissue microarrays. Whereas in SIL and SCC, the nuclei of dysplastic and cancer cells respectively were prominently stained. A similar staining pattern was reported by Nicol et al., (2012), suggesting that dysregulation of cell-cycle entry in cervical carcinogenesis was prominent in the epithelial cells (Freeman et al., 1999). There was a significant difference in MCM2 histoscore between the three histological groups, with the highest histoscore noted in HSIL compared to normal (P = 0.010). MCM2 was ubiquitously expressed across all histological groups reflected by positive staining in 6 of 9 (66%) cases of normal cervix, 29/42 (69%) in SCC, 9/10 (90%) in LSIL and 10/10 (100%) in HSIL in the tissue microarrays. Previous studies reported an increasing* MCM2* expression pattern from normal cervix to SIL and the highest in SCC (Nicol et al., 2012; Zheng, 2015; Saritha et al., 2018). *MCM2*, *3 *and *4* were also observed to be highly expressed in cancer cells compared to normal cervical epithelial cells (Ishimi et al., 2003; Amaro Filho et al., 2014). 

There are several plausible reasons for this discrepancy. Firstly, in our study, MCM2 protein expression did not correlate with its gene expression. *MCM2* gene was found to be highly upregulated in SCC group though the MCM2 protein was only highly expressed in 30% of SCC cases in the tissue microarrays. Notwithstanding the low numbers of normal cervix and SIL cases in the current study, it is known that gene expression and protein expression could be inversely linked as post-transcriptional modifications are key to the final synthesis of the protein. MCM2 protein processing including its half-life and degradation in cells may also account for the differences observed above. The MCM2 protein has been shown to function as a hexamer complex, indicating that the formation of this complex is required to ensure its activation state (Sun et al., 2014), and this was not found at the gene level. High *MCM2 *expression has been correlated with high-risk *HPV* and *p16/CDKN2A* expression in cervical cancer samples (Santin et al., 2005; Akagi et al., 2014; Zheng, 2015; Liao et al., 2018). 

The upregulated gene expression of *MCM2* in the current study may be due to the presence of HPV oncoproteins in the samples. In the natural viral life cycle of high-risk HPV infection of the cervix, the HPV E7 oncoprotein causes the inactivation of RB tumor suppressor gene in the host cells. Degradation of RB activates the CDKs to trigger E2F transcription factor leading to activation of cell proliferation pathway, which is partly regulated by the *MCM* genes involved in initiating DNA replication and cell division. This is proven in a study using HeLa cells, where HPV E7 oncoprotein binds to hyperphosphorylated RB to destabilize the RB/E2F complex (Goodwin and DiMaio, 2000). Therefore, the HPV oncoprotein indirectly causes upregulation of MCM genes and proteins. Studies on gene profiling have demonstrated upregulation of *MCM* genes as part of the DNA replication pathway in HSIL and invasive cervical cancer (Santin et al., 2005; Niu et al., 2017). Initially as a response to HPV infection, basal cervical epithelial cells are postulated to induce genes that allow the virus to replicate by evading the host cellular immune system and favour cell proliferation which ultimately transforms infected cells into LSIL (Gius et al., 2007). 

The results reaffirm MCMs as essential in DNA replication and markers of cell proliferation. The diagnostic value of MCM2 has been examined by various studies. Due to its ubiquitous nature, MCM2 is less likely to be considered on its own as a sensitive diagnostic marker for cervical cancer but shows a certain degree of promise as a concatenated detection method along with *HPV* typing, *p16 or Ki-67* biomarkers (Zheng, 2015; Liao et al., 2018). 

In conclusion, the upregulation of *MCM* genes expression in cervical carcinogenesis reaffirms MCM as a proliferative marker, whereby dysplastic and cancer cells proliferation become increasingly dysregulated and uncontrolled. The strong MCM2 protein expression in HSIL may be helpful as an added screening tool in detecting pre-cancerous cervical lesions. 

## Abbreviations

CDC6: Cell Division Cycle 6

CDT1: Chromatin licensing and DNA replication factor 1

CDK: Cyclin-dependent kinase

DNA: Deoxyribonucleic acid

FFPE: Formalin-fixed paraffin-embedded

HTA: Human Transcriptome Array

HPV : Human papillomavirus

HSIL: High-grade squamous intraepithelial lesion

LSIL: Low-grade squamous intraepithelial lesion

MCM: Minichromosome maintenance complex

RB: Retinoblastoma

SCC: Squamous cell carcinoma

SIL: Squamous intraepithelial lesion

TMA: Tissue microarray
